# Effect of nutritional education intervention to reduce anaemia during pregnancy in Dodoma City, Tanzania: protocol for a cluster randomized controlled trial

**DOI:** 10.1093/biomethods/bpab012

**Published:** 2021-06-08

**Authors:** Mariam J Munyogwa, Nyasiro S Gibore, Agatha F Ngowi, Ipyana H Mwampagatwa

**Affiliations:** 1 School of Medicine and Dentistry, University of Dodoma, P. O. Box 395, Dodoma, Tanzania; 2 School of Nursing and Public Health, University of Dodoma, P. O. Box 395, Dodoma, Tanzania

**Keywords:** anaemia, pregnant women, nutritional education, community-based intervention, dietary practices, knowledge about anaemia, haemoglobin, sulphadoxine–pyrimethamine, mebendazole

## Abstract

The objective of this study is to assess the effectiveness of community-based nutritional intervention in reducing the burden of anaemia during pregnancy. Study design will be a cluster-randomized controlled trial. Study setting will be peri-urban wards of Dodoma City. The study will have two arms (the interventional and the control arms). A total of 400 pregnant women at second trimester will be recruited. The study will consist of four phases in four months for both the interventional and the control arms namely: baseline, first and second follow-up and end-line surveys. During each phase, participants from both arms will be measured for haemoglobin concentration and assessed for gestational age, dietary practices and knowledge about anaemia. Furthermore, all participants will receive iron and folic acid supplements, sulphadoxinepyrimethamine and mebendazole tablets throughout the entire period of the study. Nutritional education will be provided to the interventional arm only during each phase. Main outcomes of the study will be changes in haemoglobin concentration, nutritional knowledge and dietary practices at each phase after the baseline survey in the interventional compared to the control arm. Descriptive statistics will be used to describe the participants. Independent and paired t-tests will be performed to make comparisons between and within groups. P-values less than 0.05 will be considered statistically significant.

**Trial registration** PACTR Registry, PACTR202007617885299. Registered on 28 May 2020.

## Introduction

Anaemia is a condition in which the number of red blood cells or their oxygen carrying capacity is insufficient to meet physiological needs [1, 2]. Anaemia can be classified into three categories: severe, where haemoglobin is <7.0 g/dl; moderate, where the haemoglobin is 7.0–9.9 g/dl; and mild, where haemoglobin is 10–10.9 g/dl [3]. According to World Health Organization, anaemia is considered to be of public health significance if population studies find a prevalence of ≥5.0% [4].

Globally, about 56% of pregnant women in low- and middle-income countries have anaemia. Sub-Saharan Africa has the highest prevalence of anaemia among pregnant women (57%), followed by South East Asia (48%) and the lowest prevalence (24.1%) in South America [5, 6]. In Tanzania, the prevalence of anaemia among women (15–49 years) is 44% in Mainland and 60% in Zanzibar [7]. The prevalence of anaemia among pregnant women in some regions of Tanzania include 68% in Dar es Salaam [8] and 47.8% in Kilimanjaro [9]. In Dodoma region, there is a scarcity of recent information on the prevalence of anaemia among pregnant women, but one past study that was conducted in Dodoma reported high prevalence (63.8%) [10].

Studies have shown several factors that can cause anaemia during pregnancy; these include micronutrients deficiencies (such as iron, folic acid, vitamin A and vitamin B_12_) and parasitic diseases, such as malaria and hookworm [9, 11–14]. Although the contribution of each of these factors to anaemia during pregnancy differs significantly by geographical location, seasons and dietary practices [15], inadequate intake of diets rich in iron is reported to be the leading cause of anaemia during pregnancy [11, 12]. Iron deficiency is the cause for 75% of all anaemia cases during pregnancy [15, 16]. Infectious diseases, such as malaria, helminth infestations and human immunodeficiency virus, are also implicated with a high prevalence of anaemia in sub-Saharan Africa [17, 18].

It has been reported that anaemia during pregnancy has negative effect on maternal and child health. These include increased maternal and perinatal mortality, increased numbers of preterm births and/or low-birth weight, impaired cognitive development in children and reduced adult work productivity [6, 19, 20]. Anaemia is also associated with intrauterine growth retardation, which is a risk factor for stunting among children of <2 years [20]. Reduction of anaemia during pregnancy can contribute significantly to achieving many of the Sustainable Development Goals (SDGs) including SDG 1 (end poverty), SDG 2 (2.2 end all form of malnutrition) and SDG 3 (3.1 maternal mortality reduction and 3.2 child mortality reduction) [21].

In Tanzania, different interventions are being implemented at health facility level to reduce the burden of anaemia during pregnancy. These include anaemia screening and treatment, supplementation of iron and folic acids (FEFO) tablets, de-worming, intermittent prophylactic treatment (IPTp) for malaria with sulphadoxine pyrimethamine (SP), free provision of mosquito nets and health education during antenatal care (ANC) visits [7]. Despite the ongoing interventions to reduce the burden of anaemia among pregnant women, the prevalence of anaemia among pregnant women in this country is still high [8–10]. This could be due to the ways in which these interventions are provided which gives opportunity to pregnant women who attend for ANC services only [7]. In case the pregnant woman fails to attend ANC clinic, will miss this opportunity and those who delay in booking their ANC visit will not receive adequate services, the lack of which may predispose them to anaemia. In Tanzania, utilization of maternal healthcare services among pregnant women is still low as it has been shown that only 24% of pregnant women had their first ANC visits in the first trimester, while 73.5% of pregnant women attended ANC in the second and third trimesters of pregnancy [7, 22]. A study conducted in Dodoma region also showed that, only 30.6% of women attended the first ANC within the first trimester of pregnancy [23]. This translates to less opportunity for receiving regular scheduled check-ups and interventions for pregnant women.

Late booking for ANC may interfere with adherence to FEFO supplementation as well as IPTp for malaria and de-worming programmes, thereby increasing the risk of anaemia in pregnancy [7]. In order to make sure that the majority of pregnant women are reached by the interventional programmes, there is a need to scale up these interventions at the community level the gap that this project aims to bridge. Moreover, there are limited interventional studies in the particular area. For instance, one study was conducted in 2002, focusing only on micronutrient-fortified beverage supplementation [10]. The current study will employ a multi-interventions module involving supplementation, IPTp for malaria, de-worming and nutritional education at the community level, where the majority of pregnant women will be reached. The aim of this study is therefore to assess a community-based nutritional education intervention to reduce the burden of anaemia among pregnant women in Dodoma City. Specifically, the objectives of this study are:

- to determine the effectiveness of community-based nutritional education intervention in reducing the prevalence of anaemia among pregnant women in Dodoma City;
- to assess the effectiveness of community-based nutritional education intervention on knowledge of anaemia (causes, signs and symptoms, effects, risk factors, prevention, dietary influences) among pregnant women in Dodoma City; and
- to assess the effectiveness of community-based nutritional education intervention on dietary intakes and practices in Dodoma City.

The results from this study will help programme planners and policy makers to plan for effective intervention strategies for reducing the burden of anaemia during pregnancy. The findings of this study will also serve as the baseline information for future scholars. Utilization of the knowledge obtained will enable pregnant women to play their role by improving their diet and adherence to maternal health services, thereby reducing maternal anaemia in Dodoma Region.

## Materials and methods

### Study area

The study will be conducted in peri-urban areas of Dodoma City. Dodoma City is the capital of Dodoma region and the national capital of Tanzania [24]. Administratively, the city is divided into 41 wards (26 urban wards and 15 peri-urban wards) with approximately a total population of 410 956 and annual population growth rate of 2.7% [24]. Women comprise ∼51% of the total population [7]. The city is inhabited by different ethnic groups although the indigenous ethnic groups are Gogo, Rangi and Sandawe [24]. The main economic activities in the city include businesses, seasonal agriculture that depends on unimodal rainfall and agro-pastoral activities. The main staple foods include maize, sorghum and millet [24]. Within the city, maternal healthcare services are provided in all healthcare facilities in order to prevent maternal-associated morbidities including anaemia. However, in remote areas the healthcare facilities are located faraway; therefore, outreach services are normally carried out on monthly basis in order to provide maternal healthcare services in those areas. Anaemia is one among the nutritional challenges affecting pregnant women within the region [10] and currently there are limited studies on anaemia among pregnant women in this area.

### Study design and population

The study design will be a community-based cluster randomized controlled trial with both control and interventional arms. Four hundred pregnant women at second trimester of pregnancy (gestational age from 13 weeks and above) living in peri-urban areas of Dodoma City at the time of data collection and willing to participate will be included in the study. Pregnant women with special medical conditions, such as epilepsy, sickle cell anaemia, diabetes mellitus, hypo- or hyperthyroidism, malignancy disease, hypertension, human immunodeficiency virus, ante-partum haemorrhage, renal disease and severe anaemia will be excluded from the study. ANC card and health worker will assist in identifying the eligible participants.

### Recruitment

Participants will be recruited by community health workers and local leaders from their households. Advertisement at the community meetings/gatherings, religious centres and Reproductive and Child Health clinics and public announcements methods will be used to mobilize the community about the study.

### Intervention

Previous studies have shown that the use of nutritional education intervention, FEFO supplementation in combination with appropriate IPTp of malaria and de-worming during pregnancy reduces the prevalence of anaemia [25]. This community-based nutritional intervention will consist of the following four components: nutritional education, FEFO supplementation, de-worming and provision of IPTp.

- Nutritional education

Nutrition education will be delivered by a research team member using a developed education material. The education material (see Supplementary file 1) has been developed by a research team after doing an intensive literature review from previous studies [26, 27]. The developed educational material has been modified and translated to Swahili language in order to suit our environment. Each pregnant woman in the interventional group will receive nutritional education. Nutrition education consists of the followings: definition of anaemia, symptoms of anaemia, causes for anaemia, effects of anaemia, behavioural and dietary risks for developing anaemia and prevention of anaemia. Nutritional education will be delivered during all three phases of the study. The first phase will be delivered at the time of baseline study where hard copy of education material will be provided to each participant for self-learning. The second phase will be followed 1 month after the first, and the last phase will be carried out 1 month after the second phase. Hard copy of training material will be provided only during the first phase (baseline survey), but the same education material will be used to train pregnant women about nutritional education for all the three phases of the study. The nutritional education training will be provided in groups of 10–11 women. Sessions will be interactive and are expected to last for ∼45–60 min.

ii. Supplementation of FEFO tablets

All participants in the interventional and control arms will receive one tablet of FEFO (200 mg of dried Ferrous Sulphate + 0.25 mg Folic Acid) supplement once daily as per ANC national guidelines [28] during the entire course of the study period. The FEFO supplements will be given on monthly dose basis starting at the baseline survey. Nurse midwifery will dispense the monthly dose of FEFO supplements to each participant. Monitoring of adherence to FEFO supplementation will be carried out on a monthly basis during follow-up visits.

iii. Malaria prophylaxis

All participants in the interventional and control arms will receive a dose of three SP (1500 mg of Sulphadoxine + 75 mg of Pyrimethamine) tablets on monthly basis as per ANC national guidelines [28] throughout the study period. The first dose will be given as soon as the nurse midwife confirms the gestation age of ≥13 weeks. The subsequent doses will be provided on monthly basis during follow-up visits. The SP tablets will be administered through the direct observed treatment method.

iv. De-worming

All participants in the interventional and control arms will receive a single dose of anti-helminthiasis as per ANC national guidelines [28]. Five hundred milligrams of mebendazole tablet(s) will be given to each participant as soon as the nurse midwife confirms the gestation age of ≥13 weeks. Mebendazole will be administered as a stat dose through the direct observed treatment method.

### Outcome measures

- Primary outcome

Primary outcome measure will be the change in haemoglobin concentration determined by blood test at baseline, first and second follow-up and end-line survey in the interventional compared with the control arm. Other variables that will be assessed along with the main primary outcome include maternal weight gain, gestational age and blood pressure.

ii. Secondary outcomes

Secondary outcome measures will include the change in nutritional knowledge and dietary practices determined by standard questionnaire at baseline, first and second follow-up and end-line surveys in the interventional compared with the control arm. Other variables that will be assessed along with secondary outcome include still birth, Appearance, Pulse, Grimace, Activity, and Respiration (APGAR) score, birth weight and new-born mortality.

### Sample size calculation and selection

The sample size will be calculated based on the formula for a randomized control study [29].

$$n=\frac{{\left\{Z\alpha \sqrt{\left[\pi o\left(1-\pi o\right)\right]}+Z\beta \sqrt{\left[\pi 1\left(1-\pi 1\right)\right]}\right\}}^{2}}{{\left(\pi 1-\pi o\right)}^{2}}$$

where *n* is the minimum sample size, *Zα* is the standard normal deviate (1.96) at 95% confidence level for this study, *Zβ* is the standard normal deviate (0.84) with a power of demonstrating a statistically significant difference before and after the experiment between the two groups at 90%. *πo* is the proportion at pre-intervention (baseline prevalence of anaemia 50%). *π*1 is the proportion after intervention (prevalence of anaemia after intervention proportion 35%).

$$178=\frac{{\left\{1.96\sqrt{\left[0.5\left(1-0.5\right)\right]}+0.84\sqrt{\left[0.35\left(1-0.35\right)\right]}\right\}}^{2}}{{\left(0.35-0.5\right)}^{2}}$$

With the adjustment for 10% attrition, the calculated sample size will be *n* = 198 (178/0.9). Therefore, a total of 200 pregnant women from each arm (interventional and control) will be recruited for the study. The study arms will be matched in the ratio of 1:1. A list of all wards will be obtained from the administrative authority of Dodoma City. From the list, wards from peri-urban areas will be listed and assigned random numbers. Simple random sampling technique will be used to select four peri-urban wards. Two wards based on geographical area will be assigned to the interventional arm and the remaining two wards to the control arm. From each ward, community health workers and local leaders will recruit eligible participants and invite them at a recommended and agreed place to conduct the study.

### Randomization

The unit of randomization will consist of wards. Wards from peri-urban areas will be grouped into two groups based on geographical area (Eastern and Western wards). A total of four wards will be randomly selected as follows: two wards from the East and remaining two wards from the West part of the city. The two groups of wards will be randomly assigned to the study arms as follows: one group to the interventional arm and another group to the control arm, respectively. This will be carried out to ensure that interventional wards are bundled together but far away from control wards in order to minimize contamination. A statistician who will not be the part of this study will do the final randomization of wards to the interventional and control arms.

**Figure 1:**
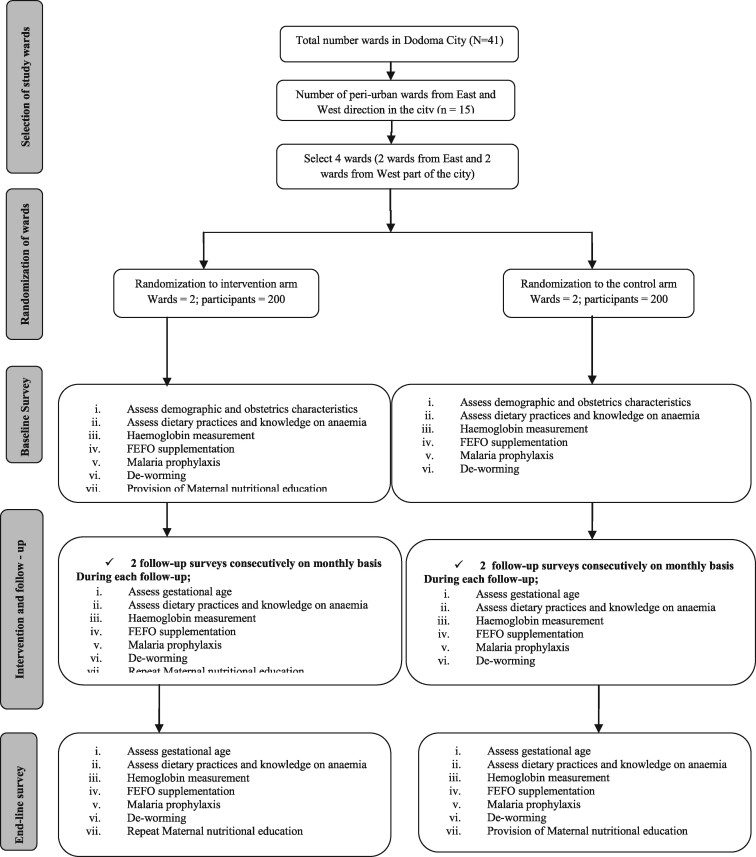
Study design and flow chart.

The flow chart of the study design is shown in Fig. 1.

### Data collection

- Baseline survey

A cross-sectional survey will be conducted in four selected peri-urban wards. Data will be collected by using a developed semi-structured questionnaire. The questionnaire has been adapted from the Food and Agricultural Organization [27] and modified to suit this study objective (see Supplementary file 2). The questionnaire consists of the six parts, namely, part 1—Demographic information; part 2—Obstetrics history; part 3—Utilization of ANC services; part 4—Knowledge about anaemia (definition, symptoms, causes, prevention, effects, behavioural and dietary risks and preventive measures); part 5—Current dietary practices; and part 6—Maternal health characteristics (anthropometric, haemoglobin and blood pressure).

Data collection will be conducted by researchers and research assistants through face-to-face interviews using the developed questionnaire. Researchers and research assistants will consist of the following professionals: nutritionists, public health specialists and registered nurses–midwife. Nutritionists and public health specialists will administer the questionnaire and provide nutrition education. Registered nurse will be responsible for pregnancy confirmation, measurements of gestational age and administering of FEFO, SP and mebendazole. Haemoglobin measurements will be conducted by the experienced laboratory scientist/technician. Haemoglobin concentration will be measured from a single drop of capillary blood via a finger prick. Prior to interview, researchers/research assistants will explain the purpose of the study to the study participants, and written or verbal informed consent will be taken from participants. Pregnant women found to have severe anaemia will be given a referral letter to a nearby health facility for further investigation and management.

ii. Intervention and follow-up surveys

Participants in the interventional and control arms will be followed up monthly for two consecutive visits. During follow-up, participants in both arms will be checked for haemoglobin concentration and continue to receive FEFO supplements, and SP as per ANC national guidelines [26]. In addition, participants in the interventional arm will receive nutritional education.

iii. End-line survey

An end-line survey will be conducted in both arms. The questionnaire filled out at baseline information will be used to collect data during end-line survey. Haemoglobin measurements will also be carried out. FEFO and SP will continue to be provided as per ANC national guideline [28].

### Measurement of variables

- Dependent variable

Dependent variable in this study will be haemoglobin concentration. The haemoglobin concentration will be classified as follows: normal haemoglobin ≥11 g/dl and anaemia <11 g/dl [4].

ii. Independent variables

The independent variables include: demographic characteristics, obstetrics characteristics, utilization of ANC services, knowledge about anaemia (definition, symptoms, causes, prevention, effects, behavioural and dietary risks and preventive measures), current dietary practices and maternal health characteristics (anthropometrics and blood pressure). Other independent variables include still birth, APGAR score, birth weight and new-born mortality.

### Data processing and analysis

Data will be cleaned, edited and coded before data analysis. Statistical Package for Social Sciences version 25.0 will be used to analyse the data. Descriptive statistics will be used to generate frequency distribution, mean, standard deviation and cross tabulation to describe the characteristics of the study participants. The comparisons between interventional and control arms will be determined using independent *t*-tests and comparisons within groups (pre/post) will be estimated using paired *t*-tests. The *P* <0.05 will be considered statistically significant. Regression analysis will be conducted to determine the predictors of haemoglobin concentration.

### Dissemination of results

The findings of this study will be presented at the University of Dodoma. Further, the findings will be communicated to the local community where this study will be conducted. Furthermore, the findings will be presented with the Ministry of Health, Community Development, Gender, Elderly and Children. In addition, we will aim to publish the findings in a suitable peer-reviewed academic journal and present these at local and international conferences.

### Ethical clearance and consent to participate

This study was submitted to the Directorate of Research, Publications and Consultancy of the University of Dodoma for ethical approval. The ethical committee has assessed and given the ethical approval for this study. Furthermore, the permission to conduct this study will be sought from the office of Dodoma Regional Administrative Secretary, District Medical Officer and ward, respectively.

The participants will have the absolute right and freedom to withdraw from the study at any time with no effect to them. Confidentiality and anonymity will be maintained by the use of code numbers on the questionnaire rather than names. Pregnant women who will be found severely anaemic will be assisted by the research assistants to seek proper treatment at the healthcare facility.

## Discussion

This will be the first cluster randomized control trial designed to evaluate the effectiveness of nutritional education intervention on anaemia among pregnant women in Tanzania. This will complement existing antenatal interventions that aim at combating anaemia among pregnant women, one of the greatest nutritional health burdens among women in Tanzania [8–10]. The cluster randomized control trial design, whereby groups of individuals are randomly allocated to different interventions, has the potential to provide unbiased estimates of the impact of interventions delivered at the community level.

Dodoma region produces various food crops, including maize, sorghum, millet, sunflower and nuts. Livestock, such as cattle, goat and chickens, is also raised and marketed within the region. However, previous studies conducted in Dodoma have reported limited awareness of the benefits of food diversity intake and thus dietary intake is often characterized by solely a single food group [30], high consumption of dark green vegetable and low consumption of meat and meat products among women [31]. This study has therefore been designed to assess the benefits of intervention that aims to provide intensive nutritional education on dietary intakes and practices, which enhance iron bioavailability, knowledge on symptoms, causes, effects and preventive measures of anaemia among pregnant women. Parallel with nutritional education, FEFO supplementation, SP and mebendazole will be provided to all study arms (interventional and control) to ensure that all individuals have received standard routine of care as per ANC national guidelines [28].

In Tanzania, utilization of maternal health care services is low [22] and such that only 24% of women attend their first ANC visits in the first trimester, while the majority (73.5%) of women attend in the second and third trimesters of pregnancy [7]. Reasons for low utilization and late attending for ANC services include living far away in remote village areas (hence fear of being harmed when going to the health care facility) and lack of money for transport [32]. This intervention is designed to be conducted in a community-based setting to enable easy and timely access of services by all pregnant women. Community healthcare workers and leaders will be involved for the mobilization of pregnant women towards the selected centres within the community.

The need for effective intervention studies addressing anaemia is particularly relevant to rural communities, where the prevalence of anaemia is highest and the majority of people are poor and less educated. This study will focus on peri-urban areas of Dodoma City. Dodoma is one of the regions with high prevalence of anaemia during pregnancy [10]. It has been reported that anaemia was associated with maternal death in 38.6% of cases in Dodoma region from 2007 to 2011 [33].

In most developing countries, micronutrients deficiencies and parasitic infections particularly malaria and hookworm have been reported to be strongly associated with anaemia during pregnancy [9, 11–13]. Furthermore, inadequate intake of diets rich in iron is reported to cause 75% of iron deficiency anaemia during pregnancy [11, 12, 15, 16]. A major strength of this trial is that it attempts to assess the contribution of nutritional education towards reducing anaemia and also to maximize early utilization of maternal health services among the pregnant women. Proper nutrition education will improve dietary intakes while using community health worker and local leaders, many more pregnant women will be identified and initiated into ANC services. It is hoped that this approach will be effective in reducing anaemia in pregnancy, thereby reducing perinatal morbidity and mortality.

The intensive nutrition education and the teaching material that will be provided to each pregnant woman in interventional and control arm at baseline and end-line survey, respectively, are expected to bring into positive changes to dietary habits in the future. If successful, such an approach could profoundly brings into the attention of the relevant authorities on how anaemia could be controlled using both, diet-focused and supplementation-focused approaches. If effectiveness in real-life conditions is determined, all the above features of the intervention could be scaled-up nationwide, and taken into consideration to improve the outcome for other conditions in pregnancy. In order to maximize policy relevance, it is essential to understand the context of the research, the related policy making processes and to engage key stakeholders. To achieve this, we intend to engage continuously with the health authorities from local to national levels.

## Supplementary data

Supplementary data is available at *Biology Methods and Protocols* online.

## Supplementary Material

bpab012_Supplementary_Data
