# Supplementary material for: Effect of nutritional education intervention to reduce anaemia during pregnancy in Dodoma City, Tanzania: protocol for a cluster randomized controlled trial
Source: Biol Methods Protoc. 2021 Jun 8;6(1):bpab012. doi: 10.1093/biomethods/bpab012 (PMC8242494; doi:10.1093/biomethods/bpab012)
Supplement: bpab012_Supplementary_Data [file bpab012_supplementary_data.zip › ADDITIONAL FILE 2 - QUESTIONAIRE- BMP - 2021 - 009.doc]

# Additional file 2: Questionnaire

**Title of the study**: Community-based maternal nutritional intervention to reduce the burden of anemia during pregnancy in Peri Urban areas of Dodoma City

**INTRODUCTION**

Date …………………………………..................

Name of interviewer..............................................

Questionnaire number………………………….

District..................................................................

Ward ………………………………....................

Street /Village…………………………..............

**Part one: Demographic characteristic of respondents**

1. In what year were you born? Year………………..
2. How old are you now? Age in completed years……….
3. What is your ethnicity? (Tribe)
1. Gogo
2. Rangi
3. Sandawe
4. Others. Specify…………………
4. What is your religious belief?
1. Christian
2. Muslims
3. Others. Specify…………………
5. What is the highest level of school you attended
1. No formal education
2. Primary education
3. Secondary education
4. Tertiary education
5. Not attended school
6. What is your occupation?
1. Unemployed
2. Employed
3. Self-employed
4. Peasant
7. What is your current marital status?
1. Married
2. Single
3. Separated/Divorced/Widowed
8. What is the highest level of school your spouse attended?
1. No formal education
2. Primary education
3. Secondary education
4. Tertiary education
5. Not attended school
9. What is the occupation of your spouse?
1. Unemployed
2. Employed
3. Self-employed
4. Peasant
10. How far from your household to the nearest health facility?
1. Within 5km
2. Above 5 km

**Part two: Obstetrics history of the participants**

11. What is your gestational age? ..............(observe the GA from RCH card………)
12. Number of pregnancies? (Gravidity)…………..
13. Number of birth? (Parity) ………………………
14. Number of live birth…………………………….
15. How old is your youngest child? ……………….
16. Have you ever had miscarried, abortion or stillbirth before this pregnancy?
    1. Yes
    2. No
17. If YES, when did such situation happen? Month………Year………
18. How many times you experience such situation…………………….

**Part three: Utilization of ANC services**

19. Have you attend ANC during this pregnancy?
    1. YES
    2. NO
20. What was your gestational age when you first attended ANC? Months……..
21. How many times haveyou attended ANC duringthis pregnancy?......................
22. Have you ever been given iron/folic acid tablets since you become pregnant?1.Yes2.No
23. IfYES, at what gestational agedid you start taking these tablets?……….
24. Have you taken any iron /folic acid tables yesterday?
    1. Yes
    2. No
25. If NO what were the reasons?.............................................
1. Due to side effect( vomiting, nausea, body weakness, heart palpitation)
2. I didn’t have the tablets
3. Negligence
4. I’m not anaemic
5. Others .Mention……………………
26. What was your source for the iron/folic acid tablet?
    1. RCH clinic
    2. Private pharmacy
    3. Others
27. During this pregnancy, have you ever taken ant-malaria drugs (SP)?
    1. Yes
    2. No
28. IfYES, at what gestational age did you start taking these tablets?……….
29. If YES, where did you get?
    1. RCH clinic
    2. Private pharmacy
    3. Others
30. How many times did you take (SP/Fansidar) during this pregnancy?............
31. During this pregnancy, did you take mabendazole drugs?
    1. Yes
    2. No
32. IfYES, at what gestational age did you start taking these tablets?……….
33. If Yes, where did you get?
    1. RCH clinic
    2. Private pharmacy
    3. Others
34. Request RCH card and record the necessary information.

| VARIABLES | SCHEDULED CLINIC VISITS (MONTHLY) | | | | | | | | |
| --- | --- | --- | --- | --- | --- | --- | --- | --- | --- |
|  | 1 | 2 | 3 | 4 | 5 | 6 | 7 | 8 | 9 |
| NO. of ANC |  |  |  |  |  |  |  |  |  |
| 200mg of ferrous sulphate and 0.25 mg of folic acid daily |  |  |  |  |  |  |  |  |  |
| The first IPTp-SP dose (three tablets) should be given as early as possible during the second trimester and at each scheduled contact, provided they are 4 weeks apart. |  |  |  |  |  |  |  |  |  |
| Mebendazole (500mg start) |  |  |  |  |  |  |  |  |  |
| Tetanus toxoid (TT) vaccination |  |  |  |  |  |  |  |  |  |

35. During (any of) your antenatal care visit(s), were you given any nutritional education?
    1. Yes
    2. No
36. How that nutritional education was provided?
    1. In a group
    2. One by one
37. How long did it take?
1. Below 30 minutes
2. More than 30 minutes
38. How many times did you receive nutritional education? ……………………
39. When did you receive nutritional educationa
    1. First ANC visit
    2. Whenever I attend ANC
    3. Other. Specify ……………………
40. Who provided health education during ANC clinic?
1. Nurse
2. Doctor
3. Other. Specify...................
41. What are the things you are taught during nutritional education session in ANC clinic? Please mention

...................................................................................................................................

..................................................................................................................................

..................................................................................................................................

..................................................................................................................................

42. During the past two weeks have ever suffered the following illness?
    1. Malaria
    2. Hookworms
    3. Others
    4. No

**Part four**: **Knowledge about anaemia (definition, symptoms, causes, prevention, effects, behavioural and dietary risks and preventive measures),**

43. Have you heard about anaemia?
    1. Yes
    2. No
    3. Don’t know/no answer
44. If Yes,what is anaemia?...............................................................................

……………………………………………………………………………………

……………………………………………………………………………………

45. Where did you heard about it
    1. Health care providers
    2. Neighbour,friend,relative
    3. Radio
    4. Television
    5. Newspapers/bronchures,pamphlets etc
    6. Others….Mention………………………
46. Can you tell among the followings what are the symptoms of anemia? (Multiple response)
1. Filling weak and tired
2. Paleness/pallor
3. Spoon nails/brittle nails (easy to break)
4. More likely to become sick
5. Unable to breath well
6. Low appetite
7. Font headache
8. Dizziness
9. Other…………Mention………………….
10. Don’t know
47. Can you tell among the following, what causes anaemia during pregnancy?
    1. Lack of iron in the diet( too little intake of of iron and folate rich food)
    2. Sickness/infection (malaria, hookworm, sikle cell, HIV/AIDS)
    3. Menstruation during pregnancy
    4. Other…….Mention
    5. Don’t know
48. Can you tell among the following how can anaemia in pregnancy be prevented?
1. Eat iron-rich foods/having a diet rich in iron
2. Eat/give vitamin-C-rich foods during or right after meals
3. Apply proper cooking technique
4. Take/give iron supplements
5. Treat other causes of anaemia (diseases and infections)
6. Other………Mention……….
7. Don’t know
49. What are the health effects of anemia during pregnancy?
1. Risk of dying during or after pregnancy
2. Low birth weight baby
3. Preterm birth
4. Other………Mention……….
5. Don’t know
50. Can you list examples of animal foods rich in iron?
1. Organ meat (Liver, Kidney,Heart)
2. Fleshmeat (Beef ,Pork ,Lamb,Rabbit,dog,Chicken,Duck)
3. Insects (Insect larvae, Senene,Grasshoppers, ants (kumbikumbi)
4. Fish and seafood (fresh fish,Dried fish,canned fish,prawns,shrimps,seafood
5. Other………Mention……….
51. Can you list examples of plant foods rich in iron?
1. Nuts (grandnuts,njugu)
2. Legumes (Beans, green peans, cow pees,soya beens
3. Cereals (maize, rice)
4. Vegetables (spinach,cassava leaves, beetroots,carrots)
5. Roots and tubers (potatoes,
52. When taken during meals, certain foods help the body absorb and use iron. What are those foods
    1. Vitamin-C-rich foods, such as fresh citrus fruits (quava, orange, lemons,apple,pawpaw, cabage, cornflower, tomatoes, green vegetables, green papers etc.)
    2. Other………Mention……….
    3. Don’t know
53. Some beverages decrease iron absorption when taken with meals. Which ones?
1. Coffee
2. Tea
3. Milk
4. Carbonated drinks
5. Other………Mention……….
6. Don’t know
54. List types of fresh citrus fruits you normally eat……………………………
55. How option do you eat
    1. Every day
    2. Weekly
    3. Monthly
    4. Don’t remember
56. When do you usually eat fresh citrus fruits?
1. Before a meal
2. During the meal
3. After a meal
4. Others
57. Do you usually drink coffee or tea?

1. Yes

2. No.

58. If Yes, how often?
    1. Every day
    2. Weekly
    3. Monthly
    4. Don’t remember
59. When do you usually drink coffee or tea?
1. Two hours or more before a meal
2. Right before a meal
3. During the meal
4. Right after a meal
5. Two hours or more after a meal
6. Others
60. Are there foods you avoid?

1. Yes

2. No

61. If YES what type(s )of food do you avoid? ……………………………..
62. If yes why do you avoid this food?………………………………………..
63. Do you crave for non food substance?

1. Yes

2. No

64. If YES which one?

1. Stones

2. Charcoal

3. Soil

4. Tooth paste

5. Raw rice

6. Other (specify).

65. Why do you think you crave / eat this substance? ……………………………
66. Do you smoke cigarette?

1. Yes

2. No

67. Do you take alcohol?

1. Yes

2. No

68. Are there any food taboos during pregnancy in your culture?

1. Yes

2. No

69. If yes. State the food taboos(s)………………………………………………..

**Part five: Current dietary practices**

70. Food consumption based on 24 hours dietary recall

| *Now I’d like to ask you to describe everything that you ate or drank yesterday during the day or night, whether you ate it at home or anywhere else. Please include all foods and drinks, any snacks or small meals, as well as any main meals. Remember to include all foods you may have eaten while preparing meals or preparing food for others. Please also include food you ate even if it was eaten elsewhere, away from your home. Let’s start with the first food or drink consumed yesterday.* | |
| --- | --- |
| 1. Did you have anything to eat or drink when you woke up yesterday? | A. Yes B. No |
| 2. If yes, specify the foods/drinks you took | ______________  ______________ |
| 3. Did you have anything to eat or drink later in the morning? | A. Yes B. No |
| 4. If yes, specify the foods/drinks you took | _______________  _______________ |
| 5. Did you eat or drink anything at the last mid-day? | A. Yes B. No |
| 6. If yes, specify the foods/drinks you took | _______________  _______________ |
| 7. Did you have anything to eat or drink during the last afternoon? | A. Yes B. No |
| 8. If yes, specify the foods/drinks you took | _______________  _______________ |
| 9. Did you have anything to eat in the evening? | A. Yes B. No |
| 10. If yes, specify the foods/drinks you took | _______________  _______________ |
| 11. Did you have anything else to eat or drink in the evening before going to bed or during the night? | A.Yes  B.No |
| 12. If yes, specify the foods/drinks you took | _______________  _______________ |

**Part six: Maternal health characteristics (Anthropometric, haemoglobin and blood pressure).** (Refer to the mothers antenatal clinic card and record for previous weight and Hb level)

71. Previous weight recorded (kgs) ……………when measured (gestational age)……………..
72. Current weight measured (kgs) …………………………….
73. Height recorded (cm) ………………………………………
74. Current MUAC measured (cm) ……………………………
75. Have ever been told that you have high blood presuure (hypertension)
    a. Yes
    b. No
76. If yes, when were you told that you have high blood pressure (hypertension)
    a. Before current pregnancy
    b. During current pregnancy
    c. Other times (mention)
77. Current blood pressure ……………………………………
78. Previous hemoglobinlevel (g/dl) ……….when measured (gestational age )……
79. Current hemoglobin level (g/dl) …………………………..
