# Supplementary material for: Effect of nutritional education intervention to reduce anaemia during pregnancy in Dodoma City, Tanzania: protocol for a cluster randomized controlled trial
Source: Biol Methods Protoc. 2021 Jun 8;6(1):bpab012. doi: 10.1093/biomethods/bpab012 (PMC8242494; doi:10.1093/biomethods/bpab012)
Supplement: bpab012_Supplementary_Data [file bpab012_supplementary_data.zip › ADITIONAL FILE 1-TEACHING GUIDE BMP - 2021 - 009.doc]

**Additional file 1**

**NUTRITIONAL EDUCATION MATERIALS TO REDUCE ANAEMIA DURING PREGNANCY**

**Definition of anaemia**

Iron deficiency anaemia is when iron levels are lower than normal in your body. During [pregnancy](https://www.ucsfhealth.org/conditions/pregnancy), your body needs more iron to make red blood cells in order to supply enough for you and your baby. However, Iron cannot be made by your body and must be absorbed from the foods you eat. In addition, your body also needs a nutrient called folate to make healthy blood cells.

**Symptoms of iron deficiency anaemia**

- Paleness of different parts of the body (skin color, white of the eyes, tongue)
- Feeling body fatigue
- Difficult beathing
- Brittle nails
- Decreased appetite
- Frequent illnesses
- Frontal headaches
- Dizziness

**Causes of anaemia during pregnancy**

- Lack/insufficient of iron and folic acid in the diet (too little intake of iron- and folate-rich foods)
- Infection/illnesses (Diseases like malaria, sickle cell, hookworms, schistosomiasis, HIV/AIDS )
- Continue to menstruate during pregnancy

**How to prevent anaemia**

- Eat foods that are rich in iron
- Eat foods that are rich in vitamin C with meals daily
- Apply proper cooking and preparation techniques for vegetables and fruits
- Take iron and folic acid (FEFO) pills daily
- Take anti-malaria and anti-worms as required
- Visit ANC services as frequent as advised

**Effects of anaemia during pregnancy**

Severe or untreated anaemia during pregnancy can increase the risk of

- Dying during or after pregnancy
- Giving birth to low birth weight baby
- Giving birth to pre-term baby
- Giving birth to still birth
- Blood transfusion

**Food rich in iron, vitamin C and Folic acid for prevention of anaemia**

**Examples of a**nimal foods high in iron

|   Fish |   Beef | Chicken |   Liver |   Sardine |   Insects |
| --- | --- | --- | --- | --- | --- |

**Examples of p**lant foods high in iron

|   Groundnuts |   Njugu |   Beans | |   Pigeon peas | |   Green peas | |   Cow peas | |   Soybeans | |  |
| --- | --- | --- | --- | --- | --- | --- | --- | --- | --- | --- | --- | --- |
|   Spinach |   cassava leaves | |   Amaranth | |   Beetroots | |   Rice | |   Maize | |  | |

**Examples of foods high in vitamin C and folic acid**

|   Orange |   Lemon | |   Guava |   Pineapple |   Pawpaw |   Green pepper | |   Sweet potatoes |
| --- | --- | --- | --- | --- | --- | --- | --- | --- |
|  | |   Spinach |   Amaranth |   Matembele |   Cabbage | |  |  |

Note:

- Iron obtained from animal foods is more absorbed in the body than iron from plant foods.
- Plant foods rich in iron should be taken with foods rich in vitamin C during meals in order to get enough iron.
  - Vitamin C help in absorption and use of iron from plant foods.
    - Example of plant meal composition that maximize the bioavailability iron to the body

**Cooking techniques to preserve vitamin C and folic acid**

- Vitamin C and folic acid can be easily lost during cooking. It is heat sensitive and may be destroyed by either high heat UV light and prolonged cooking. the following tips may help to reduce the loss of the vitamin C and folic acid:

**Consider These Tips when preparing the vegetables/fruits**

- Add 2-3 minutes close to serving time as possible to avoid loss of nutrients.
- Vegetables should be firm to the bite, pleasantly crunchy crisp) -tender (do not overcook).
- Soft vegetables, like greens, cook quickly while hard and starchy vegetables like potatoes, cook longer.
- Raw vegetables should be thoroughly washed and crisply cut in uniform pieces before adding to stew to allow for even cooking
- Vegetables should be washed before peeling to preserve vitamin C and folic acid
- Never dry vegetables under the sun.
- Don’t use baking soda/ “magadi” during cooking vegetables
- Eat vegetables/ fruits with food.

**Behavioral and dietary risk factors for anaemia**

- **Beverage that inhibit absorption of Iron**

Certain beverage when taken during meals they inhibit absorption of iron in body. Beverage such as

- Coffee
- Tea
- Beverage containing caffeine (carbonated drinks such as soda)
  - These beverages should never be taken with meals, and if must, these drinks should be taken 1-2hours after meals
- **Food pica during pregnancy**
- Food pica is craving substances with little or no nutritional value. Most pregnancy and pica related cravings involve nonfood substances such as charcoal, soil, stone, raw rice and toothpaste.
  - Stop pica as eating of such substances may lead to anaemia through causing diseases or deny the person to eat nutritious food.
- **Other factors enhance iron bioavailability includes**
  - Avoidance and restriction of some types of food
  - Don’t smoke
  - Stop alcohol drinking during pregnancy
